# Ileal Conduit versus Cutaneous Ureterostomy after Open Radical Cystectomy: Comparison of 90-Day Morbidity and Tube Dependence at Intermediate Term Follow-Up

**DOI:** 10.3390/jcm13030911

**Published:** 2024-02-05

**Authors:** Parth U. Thakker, Justin Manuel Refugia, Dylan Wolff, Randy Casals, Corey Able, Davis Temple, Alejandro R. Rodríguez, Matvey Tsivian

**Affiliations:** 1 Department of Urology, Atrium Health Wake Forest Baptist, Winston-Salem, NC 27157, USA; pthakker@wakehealth.edu (P.U.T.);; 2 John Sealy School of Medicine, University of Texas Medical Branch, Galveston, TX 77555, USA; 3 Wake Forest School of Medicine, Winston-Salem, NC 27157, USA

**Keywords:** bladder cancer, radical cystectomy, urinary diversion, ileal conduit, cutaneous ureterostomy

## Abstract

**Background**: This study aims to compare perioperative morbidity and drainage tube dependence following open radical cystectomy (ORC) with ileal conduit (IC) or cutaneous ureterostomy (CU) for bladder cancer. **Methods**: A single-center, retrospective cohort study of patients undergoing ORC with IC or CU urinary diversion between 2020 and 2023 was carried out. The 90-day perioperative morbidity, as per Clavien–Dindo (C.D.) complication rates (Minor C.D. I–II, Major C.D. III–V), and urinary drainage tube dependence (ureteral stent or nephrostomy tube) after tube-free trial were assessed. **Results**: The study included 56 patients (IC: 26, CU: 30) with a 14-month median follow-up. At 90 days after IC or CU, the frequencies of any, minor, and major C.D. complications were similar (any—69% vs. 77%; minor—61% vs. 73%; major—46% vs. 30%, respectively, *p* > 0.2). Tube-free trial was performed in 86% of patients with similar rates of tube replacement (19% IC vs. 32% CU, *p* = 0.34) and tube-free survival at 12 months was assessed (76% IC vs. 70% CU, *p* = 0.31). **Conclusions**: Compared to the ORC+IC, ORC+CU has similar rates of both 90-day perioperative complications and 12-month tube-free dependence. CU should be offered to select patients as an alternative to IC urinary diversion after RC.

## 1. Introduction

Bladder cancer is the 10th most common malignancy worldwide with more than 570,000 new cases diagnosed in 2020 [1]. A vast majority of patients present with non-muscle-invasive bladder cancer (NMIBC), but a subset of patients will advance to muscle-invasive bladder cancer (MIBC). Radical cystectomy (RC) with urinary diversion (UD) and pelvic lymphadenectomy is the first-line treatment option for MIBC and is occasionally implemented for treatment-refractory NMIBC [2,3,4]. Despite advances in robot-assisted laparoscopic surgery, there is a sparsity of high-quality evidence demonstrating superiority over the traditional open approach to RC [5].

Modern urinary diversion options include the use of small or large bowels for the creation of continent cutaneous UDs, orthotopic neobladders, and bowel conduits. There are reported short- and long-term complications associated with bowel use for urinary diversion, dependent upon the segment used and diversion performed. Early complications, including paralytic ileus, bowel obstruction, and uretero-enteric anastomotic leakage, may contribute to the high morbidity associated with RC [6]. Furthermore, the development of uretero-enteric strictures, parastomal hernia, conduit stenosis, and metabolic derangements may occur as long-term complications [7,8]. Several techniques have been implemented for the uretero-enteric anastomosis at the time of ileal conduit, including the Bricker and Wallace techniques with reported stricture rates ranging from 2 to 14% [9]. Despite the risk of the perioperative complications associated with small bowel use, the ileal conduit diversion is one of the most performed options [10].

To circumvent the use of a bowel segment, cutaneous ureterostomy (CU) was developed as a simpler urinary diversion associated with fewer complications [11]. Due to historical reports of high stenosis rates at the level of the fascia or stoma, CU has been relegated to use in frail patients or in emergent situations. These reports may overestimate the degree of CU stenosis, as these patients had neoadjuvant radiation, as was the practice pattern at that time [12]. Since the earliest descriptions of CU for UD in the 1930s, many technical modifications have been made to improve stenosis rates [13]. Creating a wider abdominal wall hiatus decreased stenosis rates to 13% and prolonged post-operative stenting for up to 3 months, leading to a decrease in stenosis rate of 4% [14]. Omental wrapping has also been described to improve blood flow to skeletonized ureters during urinary diversion, and may be used as an adjunctive maneuver [15]. Alternatively, continuous ureteral stenting has been implemented to circumvent the development of ureteral obstruction due to abdominal wall or stomal strictures [16].

While techniques for decreasing stenosis rates for cutaneous ureterostomy have improved, utilization of this modality has remained low. Compared to ileal conduit, CU has been associated with shorter operative times and lengths of stay, decreased blood loss, as well as fewer intra- and post-operative complications in elderly patients [17,18,19]. Comparative studies of single-stoma CU to ileal conduit in the modern era, however, are sparse. Herein, we sought to compare the perioperative morbidity and drainage tube dependence between patients with bladder cancer undergoing open radical cystectomy (ORC) with Wallace-type IC or single-stoma CU, to determine the viability of offering CU in an effort to decrease the morbidity of ORC+UD.

## 2. Materials and Methods

### 2.1. Study Design

From our Institutional Review Board approved, prospectively maintained, retrospective database of patients with non-metastatic bladder cancer undergoing RC, we conducted a retrospective cohort study comparing consecutive patients undergoing ORC with IC versus CU urinary diversion at a tertiary care, academic medical center between June 2020 and June 2023.

### 2.2. Study Population and Data Collection

The included patients underwent ORC with IC or CU and required a minimum of 90 days follow-up to capture perioperative morbidity. We recorded data from the electronic medical record into a password-secured, electronic database pertaining to clinicopathologic demographics, intraoperative details, hospital recovery, post-operative (post-op) follow-up, and perioperative morbidity. Patients’ comorbidity and functional statuses were reported with the following indices: the American Society of Anesthesiologists physical status classification (ASA, range: 1–5), the updated Charlson Comorbidity Index (uCCI, range: 0–24), and the Eastern Cooperative Oncology Group (ECOG, range: 0–5) [20].

### 2.3. Clinical Pathway

Pre-operative (pre-op) optimization included adequate staging with abdominopelvic cross-sectional imaging, chest imaging, laboratory studies, comprehensive internal medicine evaluation, ostomy nurse consultation, nutrition and smoking cessation counseling, and a urology clinic visit one week prior to surgery. For select patients, referral was made to a medical oncologist for the consideration of cisplatin-based neoadjuvant chemotherapy (NAC). For UD, patients were thoroughly counseled on the risks and benefits of both IC and CU for UD. In general, CU was selected by patients with advanced age, multiple prior abdominal surgeries, body mass index (BMI) < 30 kg/m^2^, solitary kidney, or patient preference after thorough pre-op discussion. At the time of surgery, if the surgeon determined that CU was not feasible, IC was performed and vice versa. Patients performed a chlorhexidine soap wash the day before and the morning of the surgery. There was no routine mechanical bowel prep. For only the patients planned to receive IC, alvimopan was administered pre-op.

Intraoperatively, the patients were induced with general endotracheal anesthesia (none received spinal or epidural anesthesia), had an orogastric tube placed (removed at the end of the case), and received subcutaneous heparin (5000 units) and prophylactic antibiotics (weight-based, intravenous cefazolin and metronidazole, modified to adjust for drug allergies). Regional nerve blocks for the anterior abdominal wall were performed prior to incision. All ORC with UD and pelvic lymphadenectomy were conducted via a mid-line, infra-umbilical incision by a fellowship-trained, urologic oncologist with a high-volume RC practice. For IC, the Wallace technique was used to spatulate the ureters and the conjoined ureteral plate was then anastomosed to the proximal end of the open ileal conduit with the distal end as a stoma at the right lower quadrant, as described by Kavaric et al. [21]. All CUs were performed using a modified Ariyoshi technique which has been previously described, involving a single-stoma CU at the right lower quadrant [22]. For patients with a solitary kidney (acquired or congenital), the CU stoma were matured ipsilaterally. Uretero-cutaneous and entero-cutaneous anastomoses were performed over a 7-French, single-J ureteral stent(s).

Postoperatively, patients were started on sips of clear fluids post-op day (POD) #0, followed by clear liquids on POD #1, and were advanced to regular, as tolerated, by POD #2. Intravenous fluid support was provided for POD #0–1, until the patient was able to sufficiently take in liquids by mouth. Mobility was encouraged to chair and ambulation with assistance starting from POD #0. Bowel regimen included daily oral senna, chewing gum, and a bisacodyl suppository until bowel movement (BM). For patients with IC, alvimopan was continued until the first BM. Pain control regimens included as-needed oral opioids (oral oxycodone, hydrocodone, or tramadol) and scheduled non-opioid analgesics (oral acetaminophen or celecoxib and intravenous ketorolac). The daily use of incentive spirometry was encouraged. Oral or intravenous anti-emetics were provided as needed. Lastly, subcutaneous heparin was continued as a thromboembolic prophylaxis and, at the time of discharge, patients were transitioned to a daily subcutaneous enoxaparin or heparin for a total 30-day course.

### 2.4. Follow-Up and Surveillance

All patients were evaluated approximately two weeks after discharge for a post-operative visit with the surgeon and ostomy nurse. Oncologic surveillance was performed according to established guidelines, with the first post-op cross-sectional imaging carried out within the first three months [2,3]. Patients with ypT2-ypT4a, ypN+ (node positive), pT3-pT4a, and pN+ disease were referred to a medical oncologist for the consideration of adjuvant therapy (nivolumab [if prior NAC], cisplatin-based regimen [if no prior NAC], or clinical trial). The decision to remove ureteral stents (with 24 h prophylactic oral antibiotics) for the tube-free trial was made at the performing surgeon’s discretion within the first three months post-op. Ureteral stents were maintained for patients with a clinically advanced disease on initial surveillance imaging (e.g., malignant external compression of ureter) or per patient preference. In cases of the need for drainage tube replacement (e.g., ureteral obstruction causing pyelonephritis or compromised renal function, ureteral anastomotic leak, or planned exchanges), a retrograde ureteral stent or percutaneous nephrostomy tube was performed by the interventional radiology team.

### 2.5. Outcomes

The 30- and 90-day morbidity were defined by the occurrence of peri-op complications, classified in severity using the modified Clavien-Dindo classification, with grades I-II considered minor, and grades III-V considered major complications [23]. The cumulative number and severity of 90-day complications were reported with the Comprehensive Complication Index (CCI, range: 0 [no complication] to 100 [death]) [24,25,26]. Tube-dependent urinary drainage was reported according to the duration of the tube-free trial (without replacement) and the frequency of tube dependence at the most recent post-op follow-up. The secondary outcomes include operative duration (hours), length of stay (LOS, days), and readmissions at 30 and 90 days post-op.

### 2.6. Statistical Analysis

Continuous data were summarized with medians and interquartile ranges (IQR), and were compared using the Mann–Whitney U test. Categorical data were summarized with frequencies and percentages and compared using Fischer’s exact test. Univariate comparisons were conducted between the two cohorts (IC and CU) on clinicopathologic variables, operative details, and perioperative morbidity (30- and 90-day: C.D. complications and CCI). For patients that underwent the tube-free trial, tube-dependence free survival was assessed with the Kaplan–Meier method and the results at 12 months post-stent removal were compared with log-rank test. Statistical analyses were performed with Prism version 9.0.0 (San Diego, CA, USA).

## 3. Results

### 3.1. Patient Demographics

During the study period, 78 patients with bladder cancer underwent RC+UD. Patients were excluded that underwent robot-assisted RC+IC/CU (10), RC with neobladder (3), RC+IC without uretero-enteric stents (3), or lacked 90-day follow-up data (6). We identified 56 patients (72%) that underwent ORC followed by UD with IC (26 of 56) or CU (30 of 56) and had a median post-op follow-up of 14 months (IC: 16 mos vs. CU: 13 mos, *p* = 0.6) (Table 1). For the IC and CU cohorts, patient demographics were similar with regard to age, male sex, race and ethnicity, comorbidity indices (ASA > 3, uCCI score, and ECOG > 1), prior abdominal surgery, baseline GFR ≥ 60, presence of MIBC on TURBT preceding ORC, and receipt of NAC. There was a significantly greater frequency of patients with BMI ≥ 30 in the IC cohort than the CU cohort (50% vs. 10%, *p* < 0.01). No patients had a prior history of upper tract urothelial cell carcinoma (UTUC).

### 3.2. Operative Details and Hospital Stay

The operative times were significantly longer for ORC+IC versus ORC+CU (4.9 h vs. 3.6 h, *p* < 0.0001, respectively) (Table 1). There were no patients that required alterations to planned UD at the time of surgery (e.g., IC to CU, or vice versa). Postoperatively, patients had a similar length of study for the IC and CU cohorts (median of four days vs. three days, *p* = 0.07, respectively). One patient in the IC cohort (4%) required concomitant radical nephroureterectomy due to intraoperative distal ureteral margin specimens with an extension of the tumor from the bladder to the distal and mid-ureter, without involvement of ipsilateral renal pelvis. Additionally, two patients in the CU cohort (7%) required either a bilateral distal ureterectomy or a radical nephroureterectomy for the distal and mid-ureteral tumors, respectively, without the involvement of the ipsilateral renal pelvis.

### 3.3. Perioperative Morbidity

In the first 30 days post-op, a total of 36 patients (64%) had a cumulative 64 complications (11 C.D. I, 35 C.D. II, 12 C.D. III, six C.D. IV, no C.D. V) and 25 patients (45%) required re-admission. For the IC and CU cohorts, the frequency of patients with one or more 30-day complications of any severity were similar (61% vs. 67%, *p* > 0.99, respectively). Additionally, the cohorts’ median 30-day CCI scores were similar (21 vs. 9, *p* = 0.8) (Table 2). The IC and CU cohorts were similar on the frequency of patients with one or more 30-day minor complications (54% vs. 67%, *p* = 0.4, respectively), one or more 30-day major complications (31% vs. 20%, *p* = 0.4, respectively), and 30-day re-admission (38% vs. 50%, *p* = 0.4, respectively). The distribution of 30-day C.D. I-V complication occurrences differed regarding the paralytic ileus managed, with a nasogastric tube placement with a higher frequency in the CU versus the IC cohort (33% vs. 4%, *p* < 0.01, respectively).

In the first 90 days post-op, a total of 41 patients (73%) had a cumulative 93 complications (12 C.D. I, 54 C.D. II, 19 C.D. III, seven C.D. IV, one C.D. V) and 30 patients (53%) required re-admission. For the IC and CU cohorts, the frequency of patients with one or more 90-day complications of any severity were similar (69% vs. 77%, *p* = 0.6, respectively). Additionally, the cohorts’ median 90-day CCI scores were similar (21 vs. 21, *p* = 0.9) (Table 2). The IC and CU cohorts were similar on the frequency of patients with one or more 90-day minor complications (61% vs. 73%, *p* = 0.4, respectively), one or more 90-day major complications (46% vs. 30%, *p* = 0.3, respectively), and 90-day re-admission (46% vs. 60%, *p* = 0.4, respectively). The distribution of 90-day C.D. I-V complication occurrences differed regarding the paralytic ileus managed, with a nasogastric tube placement with a higher frequency in the CU versus the IC cohort (33% vs. 8%, *p* = 0.02, respectively).

### 3.4. Tube-Dependent Urinary Drainage

A tube-free trial was conducted in 48 patients (86%), including all patients in the IC cohort, and excluding 8 patients in the CU cohort due to a locally advanced disease on initial surveillance imaging with evidence of malignant ureteral obstruction (n = 3), patient preference (n = 3), or death prior to the stent-free trial (n = 2) (Table 3). For patients undergoing the tube-free trial in the IC and CU cohorts, the post-op follow-up was similar (16 mos vs. 15 mos, *p* = 0.9, respectively). The IC cohort had the stents removed earlier than the CU cohort (22 days post-op vs. 75 days, *p* < 0.001, respectively) and the duration with no tube-dependence was similar (10 mos vs. 10 mos, *p* = 0.6, respectively).

**Figure 1:**
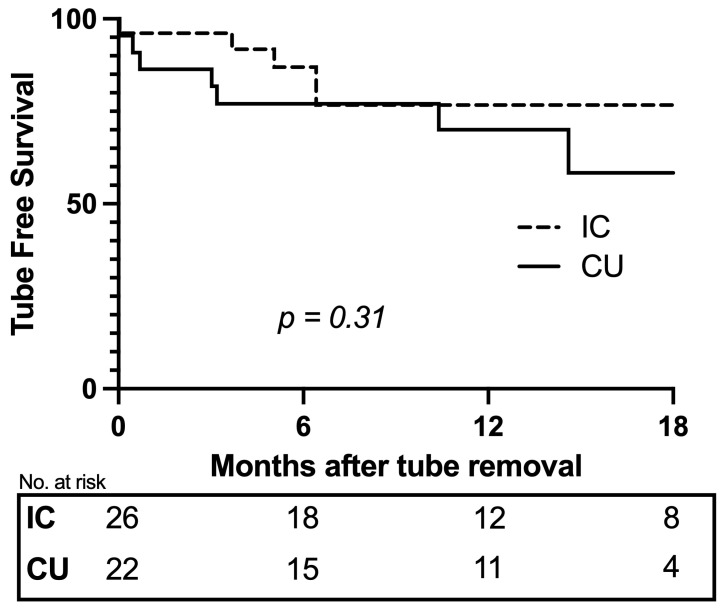
Tube free survival, following tube removal, of patients undergoing ileal conduit (IC) or cutaneous ureterostomy (CU) urinary diversion after open radical cystectomy.

In follow-up, tube replacement was necessary for 12 patients (25%) of the 48 patients that underwent the tube-free trial and occurred at a similar frequency for the IC versus CU cohorts (19% vs. 32%, *p* = 0.3, respectively). At 12 months after tube removal, tube-free survival (without replacement) was similar for the IC and CU cohorts (76% vs. 70%, *p* = 0.3, respectively) (Figure 1). The time to replacement was similar between the cohorts (5 mos vs. 3 mos, *p* = 0.7, respectively). The rationale for tube replacement is summarized in Table 3. The rate of abdominal wall stricture (identified on cross-sectional imaging) necessitating tube replacement in the CU cohort was 23%. There were no differences in indications for tube replacement. One patient in the CU cohort had a second tube-free trial conducted and failed after six months, requiring tube replacement. The remaining 11 patients continued with routine tube exchanges during the study period.

## 4. Discussion

Radical cystectomy is well known to have a high morbidity, in part due to the necessity of urinary diversion. While ileal conduit is the leading UD performed, cutaneous ureterostomy deserves consideration for the similarities in complication rates and drainage tube dependence. At intermediate-term follow-up, we have demonstrated similar 90-day perioperative morbidity and 12-month tube-free rates between patients undergoing IC and CU after open RC.

Patients with MIBC are presenting at an increasing age and those seeking RC should be counseled on efforts to optimize their recovery. For example, enhance recovery after surgery (ERAS) protocols after RC have been successfully implemented to decrease the risks of increased LOS, perioperative complications, and re-admissions [27,28]. Intraoperatively, the choice of surgical approach has a potential impact on the perioperative outcomes. Traditionally, RC has been performed via an open approach. The advent of minimally invasive surgery, such as robot-assisted RC, offers an appealing alternative to open surgery. However, there is a lack of strong evidence supporting the minimally invasive approach over the traditional open approach [5]. Considering the expedient nature of open RC, an aging population with bladder cancer might benefit from these shorter operative times. At our institution, the performing surgeon’s preference is predominantly per an open approach for our patients pursuing radical cystectomy, and this background is prudent in the assessment of our outcomes. 

The high morbidity of RC is often attributed to the necessity of urinary diversion. Selection of UD type is dependent on a composite of patient factors and surgeon experience or preference. While IC is one of the most performed UDs after RC in the United States, IC may not be a ‘one-size-fits-all’ option, especially for patients pursuing RC who have extensive medical comorbidities, precluding prolonged operative times, or prior abdominal surgeries, limiting the utilization of a bowel for diversion [10]. As an alternative to using bowel segments, CU has been shown to be a viable option, especially in elderly patients [17,18].

The tradeoffs when choosing CU over IC appear minimal given the similarities in early and late complications (30 and 90 days, respectively) in our study. The frequency of patients having any 30- or 90-day C.D. complications (C.D. I-V) were similar between the two groups, with a similar distribution of minor and major complication occurrences. When we adapted the CCI score to our cohorts to assess for the cumulative impact of the complications, there were no differences observed at 30 or 90 days post-op. In comparing to the 2021 systematic review of short-term morbidity following RC (85% IC UD) by Maibom et. al., our frequency of patients having 90-day complications appears to be similar for any complications (Maibom 59%, IC 69%, CU 77%), but differing in the distribution of C.D. I-II complications (Maibom 38%, IC 61%, CU 73%) and C.D. III-V complications (Maibom 17%, IC 46%, CU 30%) [29]. Interestingly, 90-day rates of paralytic ileus managed with a nasogastric tube were greater in our CU cohort (33% vs. 16% by Maibom et. al.), suggesting that any intraoperative bowel manipulation rather than bowel-related reconstruction may be responsible for these complications.

Urinary tract infection (UTI/pyelonephritis) is a feared complication arising from UD reported at upwards of 36% in the first 90 days, and can be attributed to compromised drainage from ureteral obstruction or leakage, as had been observed in our study [30]. Intraoperative ureteral stenting has frequently been performed to circumvent these ureteral-related complications. However, placing ureteral stents remains controversial with regard to the duration of stenting (shorter, longer, or indefinite with ongoing exchanges) and necessity of stent, which potentially increases the risk of UTIs [31,32].

The ideal duration of stenting before tube-free trial is not validated, and is typically surgeon dependent, as is in our study. For patients in the IC and CU cohorts undergoing tube-free trial, we observed similar rates of 12-month tube-free rates without the need for replacement. The impact of prolonged stenting on the tube-free rates for the CU compared to the IC cohort is unclear. Notably, of the patients undergoing the tube-free trial in the CU cohort, 23% had ureteral obstruction due to stenosis at the fascial level rather than at the stoma level, based on cross-sectional imaging, higher than the 13% reported by the retrospective series of 310 patients with CU at a median 25 months follow-up reported by Rodriguez et al. [14].

While this study potentially expands the indication for cutaneous ureterostomy, it is not without its limitations. First, our cohort study was subject to selection and information bias that is inherent to retrospective chart review. To minimize this bias, multiple members of the study group regularly reviewed the database. Second, follow-up in our cohort is relatively short, and, as such, the number of tube-dependent patients may increase over time and our follow-up duration does not allow for the evaluation of complications from drainage tube exchanges. Third, long-term renal-function changes cannot be determined based on the follow-up duration. Thus, care must be taken when counseling patients regarding the long-term complication rates related to urinary diversion. Lastly, our results have a limited generalizability as this study is reflective of a fellowship-trained, urologic oncologist with a high-volume open RC practice. Thus, there is bias with regard to the patient selection, criteria for CU versus IC, surgical technique, and the decision and timing of ureteral stent removal. Regardless of these limitations, we believe that single-stoma cutaneous ureterostomy should be offered as a viable urinary diversion with comparable tube-free and complication rates to the ileal conduit.

The main implication of our study is to encourage surgeons performing CU to add to the body of evidence in support of CU and possibly expanding the role of RC+UD in high-risk surgical candidates. Larger, multi-institutional and multi-surgeon cohorts with long-term follow-up should help surgeons to better understand perioperative morbidity, incidence of ureteral stenosis, and drainage-tube dependence in patients undergoing CU. Comparative studies should be considerate of the variations in surgical technique and the impact of RC Enhanced Recovery After Surgery (ERAS) pathways on decreasing perioperative morbidity [33]. Adapting standardized reporting for perioperative morbidity has significant utility in conducting systematic reviews and meta-analyses that may further clarify the utility of cutaneous ureterostomy [25,27].

## 5. Conclusions

Our study demonstrated that ORC with modified, single-stoma CU offers similar 12-month tube-free rates and shorter operative times with minimal compromise on 90-day morbidity compared to ORC with Wallace-type IC urinary diversion. Cutaneous ureterostomy should be presented as a viable option for urinary diversion following radical cystectomy in select patients.

## Figures and Tables

**Table 1:** Clinicopathological and perioperative details of patients receiving ileal conduit (IC) or cutaneous ureterostomy (CU) after radical cystectomy (RC).

Variable	IC	CU	*p*-Value
	n = 26	n = 30	
Demographics			
Age at RC, yr	71 (62–76)	74 (70–78)	0.10
Male sex	22 (85)	23 (77)	0.52
Caucasian race	25 (96)	26 (87)	0.36
ASA physical status > 3	4 (15)	3 (10)	0.69
uCCI score	2 (2–3)	2 (2–3)	0.31
ECOG score > 1	1 (4)	4 (13)	0.36
History of abdominal surgery	8 (31)	15 (50)	0.18
BMI ≥ 30 kg/m^2^	13 (50)	3 (10)	0.005
Baseline GFR ≥ 60 mL/min per 1.73 m^2^	13 (50)	9 (30)	0.17
Treatment details			
MIBC on TURBT Pathology	24 (92)	24 (80)	0.26
Received neoadjuvant chemotherapy	16 (61)	15 (50)	0.43
ORC operating time, h	4.9 (4.5–6.1)	3.6 (3.1–4.2)	<0.0001
Solitary kidney urinary diversion	1 (4)	1 (3)	>0.99
Length of stay, d	4 (4–6)	3 (3–6)	0.07
Non-organ confined ORC pathology (pT3-pT4 or pN+)	12 (46)	14 (47)	>0.99

Categorical data summarized with frequencies and percentages (no. [%]). Continuous data summarized with medians and interquartile ranges (median [IQR]). Abbreviations: ASA—American Society of Anesthesiologists; uCCI—Updated Charlson Comorbidity Index; BMI—body mass index; GFR—glomerular filtration rate, calculated per CKD-EPI equation; NMIBC—non-muscle-invasive bladder cancer; MIBC—muscle-invasive bladder cancer; CIS—carcinoma in situ; RC—radical cystectomy.

**Table 2:** 30- and 90-day Perioperative Clavien-Dindo Complications.

			30-Day			90-Day		
Variable	Complication	Management	IC n = 26	CU n = 30	*p*-Value	IC n = 26	CU n = 30	*p*-Value
Any (C.D. I to V)			16 (61)	20 (67)	>0.99	18 (69)	23 (77)	0.56
CCI score			21 (0–34)	9 (0–31)	0.80	21 (0–35)	21 (6–41)	0.87
Minor (C.D. I to II)			14 (54)	20 (67)	0.41	16 (61)	22 (73)	0.40
C.D. I occurrences								
	Paralytic ileus	Nasogastric tube	1 (4)	10 (33)	<0.01	2 (8)	10 (33)	0.02
C.D. II occurrences								
	Anemia	Blood transfusion	2 (8)	3 (10)	>0.99	3 (11)	3 (10)	>0.99
	Pyelonephritis	Antibiotics	3 (11)	2 (7)	0.65	5 (19)	6 (20)	>0.99
	C. difficile colitis	Antibiotics	1 (4)	1 (3)	>0.99	2 (8)	1 (3)	0.59
	SSI—superficial	Antibiotics	4 (15)	1 (3)	0.17	5 (19)	3 (10)	0.45
	SSI—deep	Antibiotics	4 (15)	3 (10)	0.69	6 (23)	4 (13)	0.49
	Wound breakdown	Antibiotics	2 (8)	1 (3)	0.59	2 (8)	1 (3)	0.59
	Malnutrition	Nutritional support	0	3 (10)	0.24	1 (4)	3 (10)	0.61
	Bacteremia	Antibiotics	0	1 (3)	>0.99	1 (4)	3 (10)	0.61
	Pneumonia	Antibiotics	0	2 (7)	0.49	0	2 (7)	0.49
	Atrial fibrillation	Chemical conversion	1 (4)	0	0.46	1 (4)	0	0.46
	Pulmonary embolism	Anticoagulation only	0	1 (3)	>0.99	1 (4)	1 (3)	>0.99
Major (C.D. III to V)			8 (31)	6 (20)	0.38	12 (46)	9 (30)	0.27
C.D. IIIa occurrences								
	Ureteroenteric anastomotic leak	Ureteral stent	2 (8)	-	-	2 (8)	-	-
	Ureteral obstruction	Ureteral stent or nephrostomy tube placement	0	3 (10)	0.24	0	3 (10)	0.24
	Intra-abdominal abscess	Abdominal drain placement	3 (11)	0	0.09	3 (11)	1 (3)	0.32
	Pelvic abscess	Pelvic drain placement	2 (8)	1 (3)	0.59	2 (8)	1 (3)	0.59
	Melena	Endoscopy	0	1 (3)	>0.99	0	1 (3)	>0.99
	Wound breakdown	Wound debridement	0	0	-	1 (4)	1 (3)	>0.99
C.D. IIIb occurrences								
	Small bowel obstruction	Exploratory laparotomy, reduction in internal hernia	0	0	-	1 (4)	1 (3)	>0.99
	Toxic megacolon	Exploratory laparotomy, bowel resection, fecal diversion	0	0	-	1 (4)	0	0.46
	Failure to thrive	Gastrostomy tube placement	0	0	-	1 (4)	0	0.46
C.D. IVa occurrences								
	Aspiration pneumonia	Intubation and mechanical ventilation	0	1 (3)	>0.99	0	1 (3)	>0.99
	Respiratory failure, COVID	ICU admission, respiratory support	1 (4)	0	0.46	1 (4)	0	0.46
C.D. IVb occurrences								
	Septic shock	ICU admission	1 (4)	3 (10)	0.61	2 (8)	3 (10)	>0.99
C.D. V occurrences			0	0	-	0	1 (3)	>0.99

Categorical data summarized with frequencies and percentages (no. [%]). Continuous data summarized with medians and interquartile ranges (median [IQR]). Abbreviations: C.D.—Clavien-Dindo; CCI—Cumulative Comorbidity Index.

**Table 3:** Tube-free trial follow-up. Only patients that had a tube-free trial were included (IC = 26 of 26, CU = 22 of 30).

Variable	IC	CU	*p*-Value
	n = 26	n = 22	
Post-op follow-up, mos	16 (7–21)	15 (10–20)	0.92
Drainage tube-dependent duration, d	22 (16–32)	75 (30–118)	<0.001
Drainage tube-free duration, mos	10 (4–18)	10 (3–15)	0.61
Change in GFR from pre-op to most recent GFR ^a^	0 (−12–10)	−4.5 (−7.5–15)	0.62
Drainage tube replaced	5 (19)	7 (32)	0.34
Time to tube replacement, mos	5 (2–6)	3 (1–10)	0.67
Reason for replacement			
Malignant external obstruction	0	1 (5)	0.46
Abdominal wall stricture	-	5 (23)	-
Uretero-enteric stricture	3 (11)	-	-
Uretero-enteric leak	1 (4)	-	-
Perirenal abscess	1 (4)	0	>0.99
Stomal stenosis	0	1 (5)	0.46

Categorical data summarized with frequencies and percentages (no. [%]). Continuous data summarized with medians and interquartile ranges (median [IQR]). Abbreviations: GFR—glomerular filtration rate; CKD—chronic kidney disease. ^a^ Excludes patients with continued stent in place.

## Data Availability

Research data are available upon request of the corresponding author.
